# How power and knowledge hierarchies affect communication in intrapartum care: findings from public health facilities in two southern Indian districts

**DOI:** 10.1186/s12884-024-06973-3

**Published:** 2024-11-25

**Authors:** Abha Rao, V. Srinidhi, Baneen Karachiwala, Sanjana Santosh, Shreelata Rao Seshadri, Sophia Thomas, Sreeparna Chattopadhyay, Anuradha Sreevathsa, Gita Sen

**Affiliations:** 1 Ramalingaswami Centre on Equity and Social Determinants of Health, Public Health Foundation of India, Bangalore, India; 2 FLAME University, Pune, India; 3 TA Pai Management Institute, Manipal Academy of Higher Education, Bangalore, India; 4 Nossal Institute of Global Health, Melbourne School of Population and Global Health, University of Melbourne, Melbourne, Australia

**Keywords:** Communication, Intrapartum care, Disrespect & abuse, Knowledge hierarchy, Power hierarchy

## Abstract

**Background:**

Effective communication is a key element of medical care; it can foster a warm interpersonal relationship, facilitate the exchange of information, and enable shared decision-making. In the context of obstetric care, it is associated with a range of positive clinical and social outcomes for mother and baby. Extant communication frameworks and respectful maternity care (RMC) guidelines emphasize the importance of effective communication during intrapartum care. Yet, studies conducted in Indian public health settings suggest that there are gaps in the implementation of RMC guidelines.

**Methods:**

As part of a larger study on disrespect and abuse in Indian public hospitals, we studied the nature of communication in the intrapartum context and the extent to which it is respectful. The study is based on interviews with 29 providers across different levels of public health facilities. Interviews were translated, transcribed, and thematically coded. We examined codes related to communication to understand what kinds of communication occur during intrapartum care and the role played by knowledge and power hierarchies. We then considered their implications for RMC.

**Results:**

We identified four types of communication that occurred in the context of intrapartum care: (a) compassionate, to comfort and support the laboring woman, (b) factual, to obtain or provide information or updates, (c) prescriptive, to obtain consent and cooperation from the woman and her family members, and (d) defensive, to protect against accusations of poor care. Knowledge and power hierarchies operated differently in each type of communication, with prescriptive and defensive communication more likely to be disrespectful than others.

**Conclusions:**

Our findings suggest that successful implementation of RMC guidelines requires greater attention to knowledge and power hierarchies, and an understanding of the ways in which they operate in a clinical setting. Integrating this understanding into guidelines, medical education, training programmes, and interventions will facilitate effective and respectful communication during maternity care.

**Supplementary Information:**

The online version contains supplementary material available at 10.1186/s12884-024-06973-3.

## Introduction

Communication is widely accepted to be an integral part of medical practice. It is particularly important in the context of obstetric or maternity care, which differs from other forms of medical care in terms of some key characteristics: pregnancy and childbirth are not considered “medical conditions” unless medical or surgical complications are involved; the duration of interaction between providers and women can vary widely: between seven to ten months, if antenatal and post-natal care is included, or hours, if only labour and birth. Furthermore, pregnancy, labour, and birth require active effort from the woman (compared to, for example, a patient undergoing a medical or surgical procedure). Finally, it is an emotionally significant event for women and their families.

Evidence shows that effective communication during intrapartum care has a significant impact on clinical and social outcomes: it is associated with an increased likelihood of vaginal deliveries compared to c-sections, shorter duration of labour, decreased use of pain relief, and decreased likelihood of babies born with a low Apgar score. Communication can contribute to a positive birth experience by helping the woman feel like she is respected and in control of making her own decisions, and can encourage bonding with the newborn [1–3].

In recent years, national and international guidelines on respectful maternity care (RMC) reflect the importance of communication. WHO recommendations for intrapartum care, for instance, call for effective communication using “simple and culturally acceptable methods” [4]. While the language of the guidelines may vary (‘person-centered care, ‘continuous support,’ etc.), they identify effective communication as a key element of care that is centered around women’s needs (dignity, confidentiality, physical relief, social-emotional comfort, freedom from harm and mistreatment) [1, 4–7]. They note, in other words, that the *how* of the birth experience is as important as the *what* [8]. Yet, the question remains: what constitutes effective communication [6]? 

Patient-provider communication can be complex and reflect asymmetries in knowledge [9, 10], but communication frameworks, such as the one developed by Ong et al. [11] or de Haes & Bensing [12], describe how communication can help patients and providers exchange information and enable care-giving. The former framework identifies how communication serves to foster an empathetic, respectful, and egalitarian interpersonal relationship [11]. The latter framework emphasizes clinical endpoints; it considers “responding to emotions as an essential function of medical communication” as it “may interfere with the other goals of the [clinical] encounter and should therefore be alleviated” (p. 289) and suggests that the patient may “participate more actively during such an encounter if they feel they are treated with respect” (p. 290). Across both frameworks, effective provider-patient communication is recognized as an important element of quality medical care and one that serves to protect patient rights, enhance patient understanding and satisfaction, increase adherence to treatment protocols, and ultimately, improve health outcomes [11, 12].

### Implementation of RMC guidelines in India

Poor quality maternity care can be a barrier to institutional deliveries in many low- and middle-income countries (LMICs) [13, 14]. Communication is an important element of quality maternity care, and one that can contribute to whether or not the woman and her family choose an institutional birth [3]. Only a few studies have examined communication with respect to obstetric care in India [15, 16], but they suggest that the above frameworks have limited relevance.

Given significant policy impetus towards institutional deliveries in many countries, it is important to understand RMC in the context of institutional intrapartum care [17]. A policy review of maternal and newborn health, family planning, and abortion policies in India and the extent to which they incorporate a person-centered approach suggests that there are multiple barriers to the implementation of guidelines that prevent the fulfilment of these goals [14].

We identify three broad themes in the literature on barriers to implementation with implications for communication:

First, public hospital settings in India and other LMICs are often resource constrained. Studies conducted in a range of hospital settings and in different parts of India, suggest that in overcrowded and understaffed obstetric facilities providers are not always able to spend time providing interpersonal care or communicating with patients [14–17].

Second, in public hospital settings, which typically provide free care to economically disadvantaged people, wide power inequalities, grounded in socioeconomic differentials, underlie the patient-provider relationship. Evidence shows that interactions in such settings can reflect and even amplify social inequalities, and can allow disrespectful communication to be ‘normalized’ as a natural characteristic of social hierarchies [18], but there is limited reflection in the RMC guidelines about the impact of such hierarchies upon communication. Provider perceptions of patients (and their families, who are routinely involved in obstetric care and decision-making) as uneducated and ignorant further contribute to indifferent communication during intrapartum care [15, 18].

Third, government programmes in India have promoted institutional deliveries, conducted by doctors and nurses with biomedical training. This has led to a decline in home births, attended by traditional birth attendants and women in the family [14]. Despite the fact that studies suggest that deliveries attended by such ‘non-skilled attendants’ in non-hospital settings are more likely to meet the standards of person-centered maternity care than those attended by nurses or doctors [19, 20], the dominant nature of biomedical knowledge in institutional settings creates a hierarchy between those who possess such knowledge and those who do not [21].

The above findings, taken together, suggest a wide knowledge and power differential or hierarchy between the provider and the patient, which can influence how providers treat pregnant and labouring women. Poor communication may reflect the impingement of such hierarchies on the provision of maternity care and may serve to create distance between providers and women [22]. Effective communication, on the other hand, can serve to reduce the asymmetry in the relationship, with implications for maternal and child health.

### Implications for communication

Indian maternity policies and guidelines such as LaQshya, a labour room quality initiative, or the country’s midwifery guidelines [23, 24] acknowledge broader discourses of RMC, and emphasize the importance of privacy, dignity, confidentiality, and courteous staff behavior. However, they do not spell out the role of effective communication, or even interpersonal care, more broadly. It is important that effective communication in the context of maternity care is actively respectful, which we define as communication that is centered around the woman’s needs, and alert to her dignity, autonomy, and social-emotional comfort.

While healthcare providers recognize that communication is critical to the patient-provider relationship, it is apparent that several factors challenge the translation of maternity care guidelines and knowledge into clinical practice [15]. These factors complicate the relationship between the provider and the woman and influence the nature of communication that occurs during intrapartum care. In such an environment, clinical interactions are likely to be constrained in their ability to foster a warm interpersonal relationship, facilitate the exchange of information, or enable shared decision-making.

We articulate, based on provider perspectives and descriptions of communication, three research questions; the first primarily descriptive, and the others analytical:

- What types of communication occur in the labour room, and what purpose do they serve during intrapartum care?
- How do power and knowledge hierarchies operate in communication between women and providers in labour room settings?
- What implications does this have for respectful communication?

## Methodology

The current study on communication during intrapartum care was part of a broader mixed methods study whose objective was to understand the drivers of disrespect and abuse (D&A) during intrapartum care in public health facilities. The study included a quantitative survey of women who had delivered in a public health facility in the year prior to the study, and in-depth interviews with obstetric care providers in public health facilities at all levels of care. The current study is based on the latter component (the survey data is being analyzed and reported separately). The study was approved by Public Health Foundation of India (PHFI)’s institutional ethics committee and conducted with requisite permission from the Government health department.

The study was conducted in 2019 in two primarily rural districts in the southern Indian state of Karnataka, that had the full range of public health facilities, ranging from village level primary health centres to district-level tertiary hospitals with attached government medical colleges and well-established undergraduate internship programmes. The facilities in which interviews were conducted were chosen in consultation with the state’s government health officials. Selected facilities were visited by the research team once before interviews were conducted. Team members introduced themselves as researchers interested in maternity care in public health facilities, and to explain the purpose of the study to the hospital administration and staff.

For the purposes of this study, we focused on providers trained in medicine and nursing who were directly involved in the provision of the technical aspects of obstetric care; as a result, we did not include other labour room staff such as cleaning staff, support staff, and ward ‘boys.’ All providers in the selected facilities agreed to participate in the study (*n* = 29, see Table 1 for details). The staff nurses and obstetrician-gynecologists were all women, while other doctors (including interns and resident trainees) included both women and men. The interns and resident trainees (referred to as junior providers) had between 1 and 5 years of experience, and the doctors (referred to as senior providers) had between 5 and 34 years of experience. The staff nurses, regardless of their experience (which ranged from 1 to 30 years), took orders from junior and senior providers.

Five members of the research team conducted the interviews, and they consisted of two medically trained doctors (VS, AS) (including one senior obstetrician-gynecologist, AS), a medical anthropologist (SC), a public health researcher (ST) and a social worker (BK). All members (four women, one man) of the research team were experienced in working on maternal health issues. Prior to interviews, they introduced themselves and included details about their training and research background. In order to reduce bias, the obstetrician-gynaecologist interviewed other experienced doctors, while remaining research team members interviewed junior doctors and staff nurses.

**Table 1:** Distribution of interviewees

Type of facility	Cadre	Interviews
Primary Health Centre (PHC)	Staff Nurse	3
	Doctor	1
Community Health Centre (CHC)	Staff Nurse	2
	Doctor	1
	Pediatrician	1
	Obstetrician/Gynecologist	2
Sub-district (*Taluka*) hospital	Staff nurse	2
	Obstetrician/Gynecologist	3
District (Teaching) hospital	Staff nurse	3
	Intern	4
	Senior Resident	2
	Obstetrician/Gynecologist	5
**TOTAL**		**29**

The semi-structured interview encouraged providers to reflect on a range of topics with respect to maternity care, such as clinical training, approaches to obstetric practice and interpersonal care, perceptions of patients, institutional and health system factors; it did not refer to D&A. Three open-ended questions focused on communication: “In your opinion, what is the role of communication in clinical practice?”; “Were you formally taught communication skills, or how and when did you pick up these skills?”; and “In what situations is communication with women and their attenders (i.e., accompanying family members) difficult? What strategies do you use to deal with these situations?”

The tool was created for a prior study on D&A conducted by our research team in urban public health facilities and was adapted to the rural health setting in the current study. One-on-one interviews were conducted in private spaces (an unused office or clinic) at the health facilities over two or three visits to each institution. Written consent was obtained for the audio-recorded interviews, which were conducted in English or the local language (Kannada) and lasted, on average, for 40 min. The interviews were translated and transcribed into English. Translations were conducted by other members of the research team and checked for accuracy by a third member.

### Analytical approach

A conventional content analysis approach, used when there is limited literature or theories on the topic of study, was applied to the data [25]. The transcripts were analyzed with the use of Dedoose Version 8.0, to examine the drivers of D&A in public hospitals.

Multiple authors (AR, VS, BK, SS & ST) took turns reading transcripts and making notes, and worked together to generate codes, review codes for consistency and clarity, expand codes as necessary, and organize them under categories. Throughout this inductive analytical exercise, the research team met regularly to ensure that the codes and categories were clear and logical; any disagreements were addressed through discussions until we arrived at a consensus. Categories included descriptions of disrespectful interpersonal care and unrecommended obstetric practices; drivers originating at the level of the health system, medical education system, and the institution; communication; socio-cultural factors, and more.

**Fig. 1:**
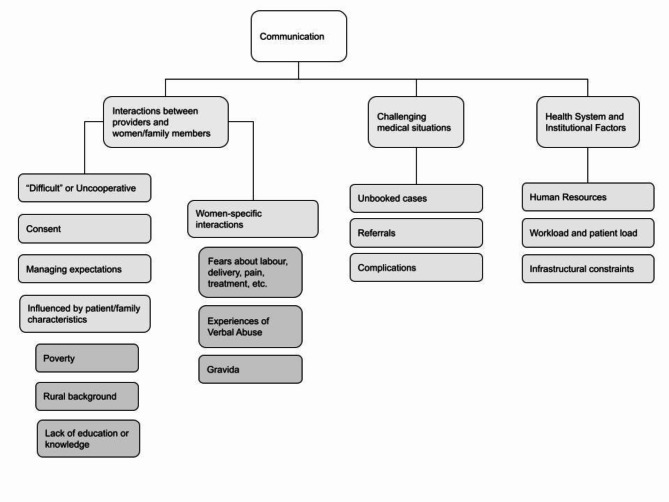
Codes related to the communication category

For the purposes of this paper, we focused on the ‘communication’ category, and noted that codes associated with this category fit under three broad areas (Fig. 1). One described (i) institutional intrapartum interactions, and others had a demonstrable (ii) proximal or (iii) distal impact on communication. In (i), codes included descriptions of provider interactions with women and/or their families (views of women/and or family members as “difficult” or uncooperative, issues around consent and managing expectations, provider perceptions of the woman’s background), and provider interactions with only the woman (about the woman’s fears, experiences of verbal abuse, perceptions based on the woman’s gravida); (ii) included codes related to challenging medical situations (unbooked cases or those with a limited antenatal history record, complications or emergencies, referrals); and (iii) codes related to broader institutional and health system factors (constraints on human resources, infrastructure, work/patient loads). We examined excerpts both within and across each area to better understand the different dimensions of communication in the context of the research questions.

## Findings

The findings are divided into four sections, starting with an overview of the different types of communication that occur in the labour room. In each section, we delve into the specific type of communication, and describe the purpose that it serves; we then pay close attention to how knowledge and power hierarchies operate within each type and examine the implications of these hierarchies on respectful maternity care.

### Overview of communication in the labour room

We identified four different types of communication, each varying in terms of the timing, purpose, individuals involved in the interaction, and tone of the communication. Our findings are organized into an overview of the key features of the four types (see Table 2). As this is a typology of communication and not a taxonomy, there may therefore be overlaps between the four types.

**Table 2:** Different types of communication

Type	Timing	Purpose	Individuals involved	Tone
Compassionate	Early stages of labour	To encourage, support the woman	Women, nurses	Kind, empathetic
Factual	Upon admission, on-going	To provide information, updates	Women, families, interns	Polite, somewhat impersonal
Prescriptive	In case of complications, emergencies	To obtain consent, cooperation	Families, doctors	Urgent, can be rude or threatening
Defensive	In case of adverse outcomes	To justify or avoid accusations	Families, doctors, hospital administrators	Tentative, self-justifying

### Compassionate communication

This kind of communication can be described as the provision of information and care with kindness. Providers engage in it to support women, particularly young women experiencing their first pregnancy, through labour and birth - to reassure her that the process will go smoothly, and comfort her through pain or fear. It is driven by providers’ own desire to provide women a positive birth experience, and also the belief that this is what women need or want. To illustrate:

*“When we ask the woman*,* ‘How was your pregnancy? Was there any problem in your pregnancy?’*,* we can see the difference in her*,* we find that she is very happy. [When we ask] ‘How many people are there? What are the foods you take?’*,* they feel*,* ‘Yes*,* this doctor is concerned about me.’ So we understand that we have to talk like this to the patient. Even when she is in labour pain we ask*,* ‘Are you comfortable? Are you finding it very difficult? Are you feeling the baby’s movements?’ Even if you just touch her*,* she will feel more happy.”* (Ob-Gyn, CHC).

This excerpt shows the provider trying to get to know the woman by asking about her accompanying family members and her food preferences, as well as finding ways to ensure her happiness, suggesting an interest in the woman’s well-being that goes beyond her medical condition. In case the woman’s medical condition is the cause of some concern, some providers seek to reassure not only the woman but her family as well:

*“If it is a difficult case*,* we make some time and talk to the family. We say the case is a bit serious*,* doctors are taking care. We tell them not to fear. [We tell the woman] we will take care of you here*,* otherwise we will send you to another higher facility*,* you have to be ready for everything. We give them courage like that.”* (Staff Nurse, District Teaching Hospital).

Although occasionally performed by doctors, this kind of communication is usually offered by the nurses during intrapartum care. As the second excerpt indicates, providers may also extend this kind of support and reassurance to family members, especially in the event of high-risk cases. Often, doctors perceive nurses to be better at providing empathetic communication, as well as better positioned to provide it, given their frequent interactions with the women, and perhaps their familiarity with the women and their families.

Compassionate communication, in which well-intentioned providers comfort, support, and encourage the woman through labour and birth, appears to be least hierarchical. However, this type of communication, although closest to what might be labelled respectful communication, is limited to the early stages of labour or upon admission to the facility, when both the woman and the provider are relatively relaxed. It does not typically sustain over the duration of labour, as other types of communication become more evident.

### Factual communication

The purpose of factual communication is to discuss the practical aspects of the patient’s condition. This could include obtaining patient history, discussing the current stage of labour or initial and continuing care, and in the case of emergencies, explaining risks and obtaining consent. It is focused on providing and gaining information and considered necessary by providers to make appropriate clinical decisions.

*“Only if we talk properly*,* will they also give us the history. They will also tell us what they require and how much. Communication is very much required.”* (Senior Resident, District Teaching Hospital).

Factual communication is particularly important in the case of women who have a limited recorded antenatal history. Interns are usually responsible for obtaining patient history when the woman is admitted to the hospital and procuring consent for routine procedures, although more senior providers step in when necessary. Providers also view the provision of on-going communication as essential for preparing the woman for potential complications:

*“[Communication] is very important. If the woman understands where she is and in what position she is in*,* she will not be anxious. She will be stable. If all the information is given to her suddenly*,* she may react differently or may go into a state of shock. If informed earlier*,* she will be mentally strong.”* (Staff Nurse, District Teaching Hospital).

Following such initial and preparatory communication, however, factual communication only continues in the event of complications. At this stage, junior providers step aside and allow senior providers to communicate with the woman and her family. Regardless of the motivation that drives factual communication or the current medical circumstances, women and their family members are only given limited information:

*“The people who come to a government hospital are uneducated*,* they are illiterate*,* they do not know anything. We’ll tell them platelet count is low*,* they will not understand*,* that hemoglobin is low*,* they will not understand. That is the problem. Even if you tell them more they will not get any extra benefit from that. If attenders are educated*,* we can tell them more*,* otherwise we only tell them what is required.”* (Ob-Gyn, District Teaching Hospital).

While this was partially a response to the limited time available to communicate with patients, there is also a pervasive belief, given the poor health literacy of the typical public hospital patient, that the women and their attenders are not capable of understanding medical information and that therefore it is quite pointless to share details. If the situation required providers to furnish additional information, there is a preference to communicate with male family members, who are perceived to be more educated and less emotional than women, or the community health worker who has accompanied the woman to the hospital, given her knowledge of hospital procedures and her connection to the woman and her family.

Providers’ ability to communicate respectfully may be further compromised by impatience with those who make repeated demands for information:

*“[Following a procedure] At that moment in time we doctors had explained it to them - the woman’s families and the attenders who are present at the scene - and calmed them down. Then these people will go and share this information with some other people. Then those people come here and again they want an explanation. Even we have limits to our patience. How many times to explain the same thing over and over?”* (Intern, District Teaching Hospital).

The tone of factual communication is usually ‘matter of fact’ - polite but impersonal. But as the second excerpt suggests, it can be respectful – the provider is thoughtful about and responsive to the woman’s needs. The third excerpt suggests that it can also be disrespectful, given the paternalism underlying the communication. The last excerpt describes the circumstances in which the providers function, which can tip communication into disrespectful territory.

Providers often control who they communicate with, and the extent to which they provide information or facilitate its exchange. These actions, grounded in assumptions about women’s knowledge and ability to understand, undermine their agency and can be considered disrespectful.

### Prescriptive communication

Prescriptive communication focuses on getting women to cooperate with the requirements of clinical care and can involve the obtaining of information or consent. This kind of communication may begin respectfully but may change in tone and intent when women or their families do not comply. For instance, to get consent for a medical procedure, providers may first explain risks and obtain consent in a factual manner; but when providers are unable to obtain information or cooperation from women, or if the urgency of the situation increases, they may use more assertive language or seek the assistance of an attender to persuade the woman to cooperate with provider requests:

*“We will ask the attenders. ‘Tell her. She is not agreeing [to a pelvic exam]. If we cannot assess*,* we cannot do anything.’ We will convince the attender*,* who in turn will convince the patient*,* who will bring and make the patient lie down on the bed. Then she will allow it.”* (Senior Resident, District Teaching Hospital).

The repeated use of the word ‘convince’ and the implicit threat of non-provision of care may be perceived as coercive, and the expectation for both the woman’s behavior and that of her family members is communicated in a demanding tone. Another strategy is to speak sternly with women and their families in order to obtain their cooperation:

*“Two out of five people we tell nicely and they understand*,* the remaining three we have to get angry for them to understand. For some people even if we tell them in a nice and loving way they will not understand. So for those people*,* we have to be a little strict*,* we have to speak a little harshly*,* then they listen”.* (Staff Nurse, PHC)

Providers may grow frustrated by the absence of key family members who can provide consent, a common practice in the Indian setting, as accompanying female family members may not feel confident giving consent, and other male members may be reluctant to do so:

*“The husbands never come here. Some attender will come and say I’m the patient’s relative and the patient also agrees*,* basically. When the patient is admitted to the labour room and she is in a serious condition*,* you speak to someone and finally you think – ‘Oh*,* he’s the husband’ - but after explaining everything*,* he’ll say ‘No*,* no*,* I’m not the husband. Her husband is standing outside.’”* (Ob-Gyn, District Teaching Hospital).

Given these conditions, already stressed providers may use even more aggressive language, such as scolding or threatening, to obtain compliance or risk consent in the event of complications or an emergency, when the need for cooperation from the woman or her family is heightened. This is illustrated in the following excerpt involving a woman with high blood pressure:

*“If the patient doesn’t listen [to instructions]*,* I will lose my temper and scold them like anything. ‘Do you want the baby or not? Why do you even marry? If your BP goes high*,* you will have convulsions and the baby will have problems. Do you understand?’ I ask such questions. Like this I will scold nicely”.* (Intern, District Teaching Hospital)

Sometimes providers use verbally abusive language to mock or belittle the woman, even in the absence of medical urgency, simply because she did not behave in the desired manner:

*“The ones who scream [from pain]*,* if we talk to them loudly*,* then they keep quiet. We tell them*,* if you scream*,* people outside who can hear you will laugh and say*,* ‘Oh*,* for a delivery she is screaming like this?’ When you take your baby and go out they will laugh at you. Then they [the women] will not scream loudly*,* they listen.”* (Staff Nurse, PHC).

This type of disparaging language is usually reported more often by those lower in the medical hierarchy. This is possibly due to their sustained interaction with the woman and her family and perhaps their rising worry and frustration in the event of an emergency; in the case of interns, it may also reflect their inexperience. Several senior providers noted that they too used to lose their patience, but experience has taught them to be more understanding.

Unlike compassionate and factual communication, prescriptive communication has a clear purpose - to obtain information, consent, or cooperation; when providers find it challenging to obtain any of these from women, they may enlist the support of other family members. If the women or their family members are reluctant to follow directives due to the lack of understanding or the absence of key family members, communication can turn disrespectful and even verbally abusive.

### Defensive communication

While compassionate, factual, and prescriptive communication occur routinely in the course of intrapartum care, defensive communication occurs only when there are severe medical complications or potentially adverse outcomes for the woman or her baby. In such cases, cautious providers sometimes take a defensive approach to communication, even from the moment of admission (in case of late referrals), to prepare the woman and her family for bad news.

To avoid blame or accusations of poor care in the wake of an adverse event, providers try to convince attenders that they have taken appropriate medical decisions. They may also attempt to transfer some of the responsibility for the woman’s condition or outcome to the woman or her family.

*“We tell the attenders that the woman will deliver after she is fully dilated. But if the woman is not pushing and not cooperating with us we call the attenders and show them what is happening. We tell them that the woman is not cooperating*,* that if something happens to the baby we are not responsible. We tell them and get their signature so they do not make noise later.”* (Staff Nurse, Taluka Hospital).

*“It was a full-term pregnancy but she came in bleeding. While treatment was going on she developed convulsions and collapsed. Her family made a lot of noise*,* shouted outside. We had to call them in and say*,* ‘When you brought her here*,* her Hb was only 4. That means you did not take care of her during her pregnancy. You didn’t give her good food with vegetables*,* grains*,* fruits and lentils. But now you are shouting?’ When we raised our voice*,* they kept quiet.”* (Staff Nurse, District Teaching Hospital).

The second excerpt describes a woman with severe anemia, a common scenario in the Indian context. As providers must routinely handle such situations, it may further contribute to their increased caution and defensiveness.

*“Sometimes what happens is that there is meconium or obstructed labour*,* or presentation is different*,* and we cannot conduct normal delivery. Majority of the attendants get aggressive*,* they say*,* ‘Initially you did not say*,* now you are saying!’ These rural people will not agree for a c-section. So that’s why we call every attender if we need to operate and explain what has to be done.”* (Ob-gyn, Taluka Hospital).

These interactions usually revolve around issues of provision of information, consent for procedures, expectations for a certain kind of birth (normal vs. c-section), and ultimately, responsibility of the involved parties. Senior providers usually handle these communications, as they have the necessary experience and authority to do so. But as the principal care providers, junior providers or staff nurses may feel the need to mount a more aggressive defense as they are more likely to face the brunt of the woman and her family’s anger or grief.

When an adverse event occurs, both providers and the woman’s family members grow aggressive and engage in defensive communication, during the event and after the actual provision of clinical care. If the adverse event draws the attention and involvement of non-family members (community members, local politicians), or includes the threat of violence or legal action, hospital administrators and medical officers may need to take such defensive measures. Unlike the other forms of communication in which there is a clear hierarchy, in these interactions it is not always clear who has the upper hand. Although defensive communication is not always disrespectful, it is rarely respectful.

## Discussion

We organize our discussion according to our key questions about communication during intrapartum care in public health facilities in southern India - first, we will examine what kind of communication occurred in these settings, then consider how they reflected knowledge and power hierarchies, and finally, whether these hierarchies had any implications for RMC.

Studies conducted in India and other LMICs have described the poor quality of communication in the context of maternity care, characterized by an absence of consent-taking procedures, limited exchange of information, and disrespectful and abusive behavior [26, 27]. Our study contributes to and expands upon this body of literature by unpacking the circumstances within which communication occurs during intrapartum care. We identified four types of communication, which varied in terms of timing, individuals involved, and tone of the interaction, with distinct knowledge and power hierarchies, and with differential impacts on respectful communication. Communication was used to comfort and support the woman (compassionate), to provide and obtain information (factual), to obtain consent or cooperation (prescriptive), and to defend against allegations of inadequate care (defensive). All of these types of communication, with varying degrees of effectiveness and respect, were underpinned by asymmetries in knowledge and power between providers and women and their families.

Most providers in our study expressed an inclination to communicate with women and their families in a respectful manner, which is consistent with the limited studies in the Indian setting. Many providers believe that sustained communication can prevent misunderstandings and even violence [15], and are accepting of simple interventions to improve communication, such as adopting the habit of addressing the patient by her name or getting verbal consent before a procedure [16]. Some of the providers in our study attempted to facilitate a positive birth experience by providing support and information during early stages of labour, following admission to the facility, but their intentions were stymied by their own perceptions and biases and the reality of obstetric care provision in public hospitals, a finding echoed in other studies [17].

Providers were often frustrated by the women, who came poorly prepared for labour and birth and typically had low literacy levels and limited knowledge about pregnancy [15, 17]. Many providers held the disrespectful belief that those with limited education lacked the ability to understand or process medical information, and it contributed to the pervasive view among providers that it was not worth providing more information than the woman and her family could understand. Providers rarely used terms such as “discuss” or “speak with,” and were more likely to say that they had to “convince” women and their attenders, or make them “listen” or “understand.” However, providers did not view their interactions as disrespectful as it was motivated by the intention of ensuring quality clinical care.

In fact, some providers viewed it as a kindness to limit how much information they provided, so as not to burden or confuse women and their families with information that they may find challenging to process. This finding is consistent with other studies that show that the term ‘communication’ was used interchangeably with ‘counseling’ or ‘guiding’ as providers were driven by a sense that women and their families did not always know what was best for them [15]. This attitude comes from a deep rooted institutional, epistemological, and cultural belief that providers know and can make better decisions than the women themselves.

Communication in labour rooms often consisted of attempts to obtain the woman’s or her family’s cooperation, or to defend themselves against accusations of poor care in the event of complications. In such cases, communication became ineffective or even disrespectful. Many providers believed that women and their families would not take medical advice seriously unless scolded or spoken to sternly. The urgency of medical emergencies overwhelmed women and their families, when asked to make decisions in the absence of information, knowledge, or meaningful choices. Providers, stressed by professional ethical demands and the risk of legal liabilities, sometimes shouted or threatened in order to obtain consent or cooperation.

Providers at all levels were involved in all four types of communication, but in both prescriptive and defensive communication, when disrespect is most evident, junior providers and staff nurses were more likely to report engaging in disrespectful and abusive behavior than senior providers. This may be due to a range of reasons: their sustained interaction with the woman and her family from admission onwards, allowing familiarity to develop; perceived (or actual) responsibility for medical emergencies and any adverse events, as the immediate care providers; and relative inexperience (in the case of junior providers) providing clinical care and interpersonal care, which may result in panicked and therefore disrespectful responses.

Medical emergencies and adverse events appeared to heighten the awareness of hierarchies. In some such medical situations, women’s family members aggressively – and even disrespectfully – pushed back against the providers, and providers often responded in an understandably defensive and self-justifying manner. This kind of interaction has become distressingly common in Indian hospitals; almost 75% of doctors report that they have been the target of verbal or physical threats by patients and their attenders. While providers blame the unreasonable expectations of the patients and family members and the deficiencies of the healthcare system for these situations [28], our findings suggest that the interactions between providers and the women and their attenders is more complex and nuanced.

All of these interactions occurred in the face of broader health system and human resource constraints, which contributed to heavy patient loads [14]. In this context, driven by the evident belief that they could either provide respectful care *or* ensure a good clinical outcome, many providers felt that spending time communicating with patients was an additional challenge to the provision of clinical care, and one that they did not feel they could fulfill [15]. Although clinical practice and caregiving should ideally be inseparable, the separation of the two has become ingrained in medical settings with the former being performed by doctors and the latter being left to nurses [29]. Many senior providers felt that their limited time was better utilized in ensuring favorable clinical outcomes, and suggested that lower-level providers or even a dedicated cadre of workers take on the responsibility of communicating with and counseling women and their families.

In summary, key elements of respectful communication - centering the woman, enabling the sharing of information and decision-making - were not visible in the study setting. Women were rarely involved in any decision-making or subsequent interactions. Equally absent was any meaningful two-way communication or dialogue between the woman and her family, and the providers. In general, there was very limited and sporadic communication, with either the woman or her family. When it did occur, it rarely served to take the woman’s needs or preferences into account, inform the woman or her family, or involve them in any decision-making. This is consistent with other studies that suggest that communication in labour rooms, when it occurs, does little to support the woman’s autonomy or agency, or address underlying knowledge and power asymmetries [15, 16].

### Research implications

Our findings suggest that researchers need to closely examine not only the circumstances, but also the language of communication. For instance, providers may characterize their escalating attempts to obtain cooperation as necessary and respectful, but women and their families may describe the same interaction as a coercive and disrespectful. Similarly, provider perceptions of whether a patient or the medical situation is ‘difficult’ may influence how they respond to or interact with the woman. Better understanding the drivers of language in the labour room can further refine our knowledge of D&A.

### Policy recommendations

These findings have implications for pre-service medical training, in-service clinical practice, and community engagement programmes.

For example, while the current undergraduate medical curriculum incorporates a module focused on attitude, ethics, and communication (AETCOM), it focuses mostly on emergency, tertiary, and end-of-life care. However, our study points to expanding these discussions to deliberate upon issues related to women’s health or maternity care, such as the principles that underlie RMC, challenges to its provision, and methods to overcome these challenges. The curriculum must directly teach methods to engage in respectful communication and should also reflect upon the importance of communication to maternal and child health and wellbeing,

Similarly, the labour room quality improvement programme, LaQshya, currently emphasizes obstetric care procedures and infrastructural reforms. Our findings indicate a need to expand the approach here too: to give sufficient attention to interpersonal care, and to adequately equip doctors to be respectful in the face of challenging work environments. Also, the LaQshya initiative should pay sufficient attention to the forces that underlie D&A, as current guidelines, in their form and function, are limited in their ability to ensure respectful care [30].

Additionally, we see opportunities to innovate community health programmes that can, to some degree, address health system challenges. For example, community health workers can be trained to lay the groundwork at the household level about the labour and birthing process, and medical providers can clarify any doubts or bridge gaps in knowledge during routine antenatal care visits. Taking these steps over the course of the pregnancy can improve the quality of communication between providers, women and their families during intrapartum care.

### Limitations

The data reported in this paper was collected five years ago; however, the findings remain relevant, as, despite the implementation of interventions that are intended to improve patient care, such as AETCOM or LaQshya, the essence of how maternity care is delivered remains unchanged.

The study examined communication in labour wards and rooms solely from the perspective of obstetric care providers. Comparing these perspectives with women’s perceptions or experiences, or researcher observations would undoubtedly have contributed to a richer analysis than we are able to provide. Nevertheless, the present analysis unpacks the logic and assumptions that underpin communication from the perspective of those who wield knowledge and power in clinical encounters.

## Conclusion

Knowledge and power hierarchies can determine the amount of information shared, shape the manner in which communication occurs, or devalue the role of communication in supporting clinical outcomes. The D&A literature has largely focused on observable behavior, and interventions have focused on preventing such behaviors and practices in the labour room, but our findings show that respect and disrespect can operate in more subtle ways.

Patient-provider communication frameworks and RMC guidelines represent a gold standard of communication. However, existing frameworks on patient-provider communication [11, 12, 31] and international guidelines on intrapartum care need to fully reflect the kind of interactions that occur in India (and other LMICs). In these settings, power and knowledge asymmetries rooted in deep social and economic inequalities operate alongside under-resourced institutional settings and heavy patient loads and drive disrespectful and abusive communication practices in labour rooms. We therefore believe that a systematic adaptation of guidelines, whether educational or programmatic, requires a better recognition of these hierarchies and center women’s voices and agency. Interventions must advance a view of effective communication that enables sharing of information and decision-making and acknowledges and addresses knowledge and power hierarchies. This can foster a clinical environment that reduces asymmetries, advances women’s agency and rights, protects women and providers, and ultimately, improves clinical outcomes.

## Electronic supplementary material

Below is the link to the electronic supplementary material.

Supplementary Material 1

## Data Availability

De-identified transcripts are available from the corresponding author upon reasonable request.
